# The Association between Circadian Syndrome and Frailty in US adults: a cross-sectional study of NHANES Data from 2007 to 2018

**DOI:** 10.1007/s40520-024-02745-3

**Published:** 2024-05-07

**Authors:** Lirong Sun, XingWei Huo, Shanshan Jia, Xiaoping Chen

**Affiliations:** 1 Cardiology Department, West China Hospital, Sichuan University, Chengdu, 610041 Sichuan Province People’s Republic of China; 2 Department of Internal Medicine, The Affiliated Hospital of Xizang Minzu University, Xianyang City, Shaanxi Province 712000 People’s Republic of China

**Keywords:** Circadian syndrome, Metabolic syndrome, Cross-sectional study, Frailty, NAHNES

## Abstract

**Purpose:**

Frailty and Circadian Syndrome (CircS) are prevalent among the elderly, yet the link between them remains underexplored. This study aims to examine the association between CircS and frailty, particularly focusing on the impact of various CircS components on frailty.

**Materials and methods:**

We conducted a cross-sectional analysis using data from the National Health and Nutrition Examination Survey (NHANES) spanning 2007 to 2018. The 49-item Frailty Index (FI) was employed to assess frailty. To understand the prevalence of CircS in relation to frailty, we applied three multivariate logistic regression models. Additionally, subgroup and interaction analyses were performed to investigate potential modifying factors.

**Results:**

The study included 8,569 participants. In fully adjusted models, individuals with CircS showed a significantly higher risk of frailty compared to those without CircS (Odds Ratio [OR] = 2.18, 95% Confidence Interval [CI]: 1.91–2.49, *p* < 0.001). A trend of increasing frailty risk with greater CircS component was observed (trend test *p* < 0.001). Age (*p* = 0.01) and race (*p* = 0.02) interactions notably influenced this association, although the direction of effect was consistent across subgroups. Sensitivity analysis further confirmed the strength of this relationship.

**Conclusion:**

This study identifies a strong positive correlation between CircS and frailty in the elderly. The risk of frailty escalates with an increasing number of CircS components. These findings highlight the intricate interplay between circadian syndrome and frailty in older adults, offering valuable insights for developing targeted prevention and intervention strategies.

**Supplementary Information:**

The online version contains supplementary material available at 10.1007/s40520-024-02745-3.

## Introduction

Frailty, a common geriatric syndrome, is characterized by diminished multi-system physiological functioning and increased susceptibility to stressors[1, 2]. It has garnered significant attention due to its association with adverse health outcomes, including frequent hospitalizations and increased mortality risk [3]. Frailty prevalence ranges from 12 to 24% among individuals over 50 and rises sharply with age[4, 5]. Notably, recent research indicates that frailty is a dynamic condition [6], potentially reversible through effective interventions [2], underscoring the importance of identifying its risk factors [7].

There is a well-established correlation between metabolic syndrome (MetS) and frailty in older adults[8, 9]. Sleep disturbances and depression, often co-occurring with MetS[8, 10], are also recognized contributors to frailty[11, 12]. Despite these known associations, previous studies have rarely examined these factors concurrently.

Our understanding of metabolic syndrome (MetS) and its associated health complications has been significantly enhanced with the advent of the Circadian Syndrome (CircS) concept. CircS is increasingly recognized as a key contributor to MetS [13]。. The diagnosis of CircS is established by the presence of at least four chronic conditions, including hypertension, dyslipidemia, increased waist circumference, diabetes, short sleep duration, and depression [13]. Recent research suggests that CircS may be a more robust predictor of cardiovascular diseases [13, 14] and stroke [15] than MetS alone, thereby underscoring its potential role in increasing the risk of frailty. Additionally, disruption in circadian rhythms can affect cognitive behaviors such as attention, alertness, higher-order executive functions, and memory [16]. Studies show that individuals with Circadian Rhythm Syndrome (CircS) are at a significantly higher risk of cognitive impairment compared to those without CircS [17]. Given the critical role circadian rhythms play in cognitive functioning, CircS could exacerbate existing cognitive decline or increase susceptibility to dementia in individuals[17, 18]. This connection underscores the importance of understanding the comprehensive effects of CircS on the health and functional status of older adults, emphasizing the need for targeted interventions to mitigate these impacts. However, the direct link between CircS and frailty in older adults remains uncertain. This study aims to investigate the association between CircS and frailty in the elderly. By examining these complex relationships, we aim to deepen our understanding of the underlying causes of frailty in old age, providing critical insights for the development of preventive and therapeutic strategies.

## Materials and methods

### Data sources and subjects

The National Health and Nutrition Examination Survey (NHANES), conducted by the Centers for Disease Control and Prevention, is a cross-sectional survey that periodically updates data on the health and nutritional status of the U.S. population. This study encompasses six consecutive survey cycles from 2007 to 2018, including 2007–2008, 2009–2010, 2011–2012, 2013–2014, 2015–2016, and 2017–2018. Data collection methods comprised questionnaires, interviews (both telephone and in-person), physical examinations, and laboratory tests. Interviews typically took place in participants’ homes, while physical examinations and blood sample collections [19] were conducted at mobile screening centers.

**Fig. 1:**
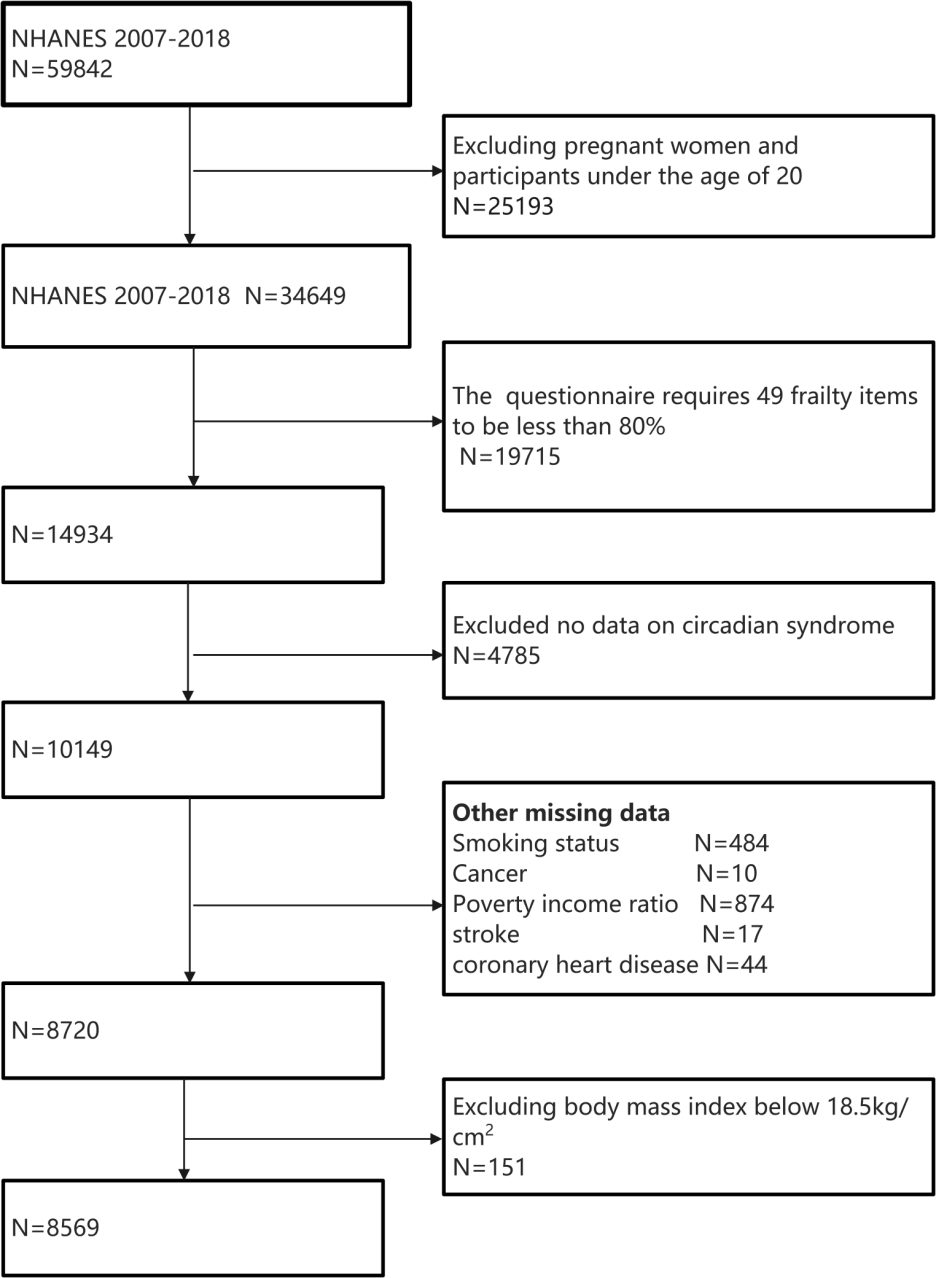
Flowchart for selecting the right participants

Informed consent was obtained from all participants, and the study received ethical approval from the National Center for Health Statistics Research Ethics Review Board (ERB). For our analysis, we initially selected data from 59,842 individuals from the NHANES database spanning 2007 to 2018, focusing on adults aged 20 years and older. To ensure the integrity and completeness of our data, we excluded pregnant individuals and cases with incomplete data sets. Ultimately, our final analysis included 8,569 participants (Fig. 1).

https://wwwn.cdc.gov/nchs/nhanes/.

### Assessment of frailty indiex

To ensure the accuracy of frailty diagnosis in our study, we employed stringent data inclusion criteria. We included participants who responded to at least 80% of the items on the frailty index, aligning with previous research indicating that a reliable analysis requires a substantial number of variables (approximately 39) for validity[20, 21]. Frailty was quantified using a deficit accumulation approach. We computed the frailty score by summing specific deficit items and then dividing this sum by the total number of considered items, resulting in a score ranging from 0 to 1. This score reflects the severity of frailty, where 0 represents no deficit and 1 indicates a complete deficit.

For analytic purposes, we transformed this continuous score into a binary variable. Consistent with prior studies, we designated a frailty index of 0.21 or higher as indicative of frailty[22, 23]. The frailty index calculation followed the standardized methodology proposed by Searle et al., encompassing 49 criteria that capture various aspects of frailty. These criteria include daily activity levels, cognitive function, physical performance, depressive symptoms, overall health status, the prevalence of chronic diseases, laboratory test results, and healthcare utilization. A comprehensive breakdown of these criteria is available in Supplementary Table 1.

### Evaluation of circadian syndrome

The diagnosis of Circadian Syndrome (CircS) is established by the presence of four or more of the following components, as delineated in prior studies[13, 24]: a. Elevated triglycerides (≥ 150 mg/dL) or usage of lipid-lowering medications; b.Waist circumference of ≥ 102 cm in men and ≥ 88 cm in women. c. Elevated blood pressure (diastolic ≥ 85 mm Hg or systolic ≥ 130 mm Hg) or the use of antihypertensive medications. d. Decreased high-density lipoprotein (HDL) cholesterol (< 50 mg/dL in women and < 40 mg/dL in men) or the use of lipid-lowering medications.e. Reduced sleep duration (≤ 6 h per day). f. Elevated fasting blood glucose (≥ 100 mg/dL) or the use of antidiabetic medications. g. Depressive symptoms, as determined by the Patient Health Questionnaire-9 (PHQ-9). Individuals meeting or exceeding this threshold of four components are classified as having circadian syndrome.

#### Covariates

Baseline data on frailty and Circadian Syndrome (CircS) were primarily gathered from questionnaires. The continuous variables included age (≥ 20 years), Body Mass Index (BMI), and the Poverty-to-Income Ratio (PIR). The categorical variables encompassed age, sex, race, PIR, educational level, alcohol consumption, smoking status, and history of cancer, coronary heart disease, or stroke. Specifically, PIR was categorized into three groups: ≤1.3, > 1.3 to ≤ 3.5, and > 3.5%. Age was stratified into quartiles as follows: 20–55, 56–63, 64–72, and ≥ 73 years. Racial categories included Other Hispanic, Mexican American, Non-Hispanic Black, Non-Hispanic White, and Other Races. Smoking status was classified as nonsmoking (individuals who have smoked ≥ 100 cigarettes in their lifetime but do not currently smoke, and those who have smoked < 100 cigarettes in their lifetime) and current smoking (individuals who have smoked ≥ 100 cigarettes in their lifetime and currently smoke either every day or on some days). Alcohol consumption was defined as < 12 or ≥ 12 drinks per year. Educational attainment was divided into five categories: less than high school, high school graduate, and more than high school diploma. Data on alcohol consumption had some missing values.

### Statistical analyses

In all analyses, we applied the sampling weights as recommended by the Centers for Disease Control and Prevention (CDC). Continuous variables were reported as mean ± standard error (SE), and categorical variables as number and percentage (n, %). Analysis of Variance (ANOVA) was used for assessing correlations among continuous variables, while chi-square tests were utilized for comparing categorical variables. Participants were categorized into two groups based on their Circadian Syndrome (CircS) diagnosis. We developed three multivariate logistic regression models to analyze the association between CircS and frailty, and also examined the relationship between the number of CircS components and frailty. The first model was unadjusted. The second, a minimally adjusted model, accounted for age, gender, and race. The third, a fully adjusted model, controlled for age, sex, race, BMI, education, PIR, smoking status, coronary heart disease, alcohol consumption, stroke, and cancer history. Additionally, we conducted sensitivity and interaction analyses to explore the effects of covariates on the CircS-frailty association. This involved incorporating stratified terms into the regression models. All statistical analyses were performed using R version 4.3.2 (http://www.R-project.org, R Foundation). A p-value of less than 0.05 was considered statistically significant.

## Results

### Baseline characteristics of participants

Among the 8,569 participants included in the NHANES 2007–2018 cycle, distinct baseline characteristics emerged, particularly when comparing groups based on Circadian Syndrome (CircS) status (Fig. 1 outlines specific inclusion and exclusion criteria). Table 1 highlights that individuals with CircS were typically older (mean age 61.84 ± 0 years), and a significantly higher prevalence of frailty was observed in this group (58.39%). Additionally, these patients were predominantly female (55.30%), exhibited higher average BMI (32.46 ± 0 kg/m²), and had greater rates of smoking (57.08%), coronary artery disease (11.25%), alcohol consumption (15.12%), and stroke (8.41%) compared to their non-CircS counterparts. In contrast, the proportions of individuals with higher income (as measured by the Poverty-to-Income Ratio, PIR, 2.57 ± 0) and higher education levels (beyond high school graduation) (49.61%) were lower in the CircS group. There was no notable difference in cancer prevalence between the two groups. Furthermore, Table 2 indicates a higher prevalence of CircS among frail patients.

**Table 1:** Characteristics of participants by categories of circadian syndrome: NHANES 2007–2018

Characteristic	Overall	Non-circadian syndrome	Circadian syndrome	*P* Value^3^
	*N* = 8569 (100%)^2^	*N* = 4542 (56%)^*2*^	*N* = 4027 (44%)^*2*^	
age (years)	60.32 ± 0.27	59.12 ± 0.27	61.84 ± 0	< 0.001
<60	35.11%	36.21%	33.70%	
≥60	64.89%	63.79%	66.30%	
PIR	2.76 ± 0.05	2.91 ± 0.05	2.57 ± 0	< 0.001
<1.3	25.57%	23.05%	28.76%	
1.3–3.5	38.52%	36.96%	40.50%	
>=3.5	35.91%	39.99%	30.74%	
BMI	29.70 ± 0.12	27.53 ± 0.12	32.46 ± 0	< 0.001
<25	24.91%	36.55%	10.10%	
≥25	75.09%	63.45%	89.90%	
frality_score	0.21 ± 0.003	0.16 ± 0.003	0.27 ± 0	< 0.001
Frality	41.28%	27.79%	58.39%	
RACE				0.001
Mexian American	4.87%	4.47%	5.37%	
Other Hispanic	4.06%	3.84%	4.34%	
Non-Hispanic Wite	75.43%	77.23%	73.15%	
Non-Hispanic Black	10.37%	9.01%	12.10%	
Other Race	5.27%	5.45%	5.05%	
**Gender**				0.013
Females	53.39%	51.88%	55.30%	
Males	46.61%	48.12%	44.70%	
**Education level**				< 0.001
Less than high school	20.53%	17.51%	24.37%	
High school or GED	24.97%	24.13%	26.03%	
Above high school	54.50%	58.35%	49.61%	
Smoke				< 0.001
YES	53.59%	50.84%	57.08%	
NO	46.41%	49.16%	42.92%	
drink	13.61%	12.42%	15.12%	0.006
cancer	18.99%	18.37%	19.78%	0.2
CHD^1^	7.61%	4.74%	11.25%	< 0.001
stroke	6.06%	4.20%	8.41%	< 0.001

^1^ CHD: Coronary_heart_disease

^*2*^ Mean ± standard error for continuous; n (%) for categorical

^*3*^ Wilcoxon rank-sum test for complex survey samples; chi-squared test with Rao & Scott’s second-order correction

**Table 2:** Characteristics of 8569 participants in NHANES between 2007 and 2018

Characteristic	Overall *N* = 8569(100%)	Frailty ≤ 0.21	Frailty>0.21	*P* Value3
		*N* = 4746 (59%)2	*N* = 3823(41%)2	
age (years)	60.32 ± 0.27	60.25 ± 0.33	60.42 ± 0.37	0.500
< 60(%)	35.11	29.22	43.48	
≥ 60(%)	64.89	70.78	56.52	
PIR	2.76 ± 0.05	3.10 ± 0.05	2.28 ± 0.05	< 0.001
<1.3(%)	25.81	19.11	35.34	
1.3–3.5(%)	38.28	36.42	40.92	
≥3.5(%)	35.91	44.47	23.74	
frailty_score	0.21 ± 0.003	0.12 ± 0.001	0.34 ± 0.002	< 0.001
BMI	29.70 ± 0.12	28.55 ± 0.11	31.35 ± 0.20	< 0.001
<25(%)	24.91	28.62	19.58	
≥25(%)	75.09	71.38	80.42	
CircS_group				< 0.001
<4(%)	55.91	68.76	37.64	
>=4(%)	44.09	31.24	62.36	
CircS				
<4(%)	77.26	87.54	62.63	
4(%)	14.31	9.37	21.34	
5(%)	6.88	2.63	12.92	
≥ 6(%)	1.55	0.46	3.10	
RACE				< 0.001
Mexian American(%)	4.87	4.77	5.00	
Other Hispanic(%)	4.06	3.58	4.75	
Non-Hispanic Wite(%)	75.43	77.71	72.19	
Non-Hispanic Black(%)	10.37	8.50	13.04	
Other Race(%)	5.27	5.44	5.02	
Gender				< 0.001
Females(%)	53.39	48.63	60.15	
Males(%)	46.61	51.37	39.85	
Education level				< 0.001
Less than high school(%)	20.53	15.88	27.16	
High school or GED(%)	24.97	23.54	27.01	
Above high school(%)	54.50	60.59	45.83	
Smoke				< 0.001
YES(%)	53.59	49.13	59.94	
NO(%)	46.41	50.87	40.06	
Drink(%)	13.61	12.52	15.16	0.002
Cancer(%)	18.99	16.39	22.70	< 0.001
CHD^*1*^(%)	7.61	3.85	12.95	< 0.001
Stroke(%)	6.06	2.48	11.14	< 0.001

^*1*^CHD: Coronary_heart_disease

^*2*^ Mean ± standard deviation for continuous; n (%) for categorical

^*3*^ Wilcoxon rank-sum test for complex survey samples; chi-squared test with Rao & Scott’s second-order correction

### Multivariate regression analysis

We performed multivariate regression analyses and present the effect sizes for the association between CircS and frality in Table 3. The results showed that in Model 1, uncorrected for any covariates, the hazard ratio for developing frailty in the CircS group versus the non-CircS group was 2.68 (95% CI 2.37–3.03, *p* < 0.001). In Model 2, corrected for race, age, and sex, the hazard ratio for developing frailty in the CircS group versus the non-CircS group was 2.66 (95% CI 2.36–2.99, *p* < 0.001). Model 3, after adjusting for all covariates, had a hazard ratio of 2.18 (95% CI 1.91–2.49, *p* < 0.001) for the occurrence of frailty in the CircS group versus the non-CircS group. In addition, multifactorial logistic regression analysis under stratified conditions, corrected for all covariates, showed that for CirS, the individual components were grouped as < 4, 4, 5, and ≥ 6. Hazard ratios for the occurrence of frailty were 2.36, 4.84, and 7.25, respectively, with an increase in the CirS component compared to CirS < 4. This indicates that the risk for the occurrence of frailty was on the rise with an increase in the CirS component, with a trend towards an increase in the risk of frailty. The trend test *p* < 0.001 suggests that the increasing trend is statistically significant.

**Table 3:** Association of circadian syndrome with the prevalence of frality

	Model 1			Model2			Model 3		
Characteristic	OR1	95% CI	*p*-value	OR1	95% CI	*p*-value	OR1	95% CI	*p*-value
CircS									
NO	Ref			Ref			Ref		
YES	2.68	2.37, 3.03	< 0.001	2.66	2.36,2.99	< 0.001	2.18	1.91,2.49	< 0.001
Components of circadian syndrome									
<4	Ref			Ref			Ref		
4	3.18	2.66, 3.81	< 0.001	3.13	2.62, 3.75	< 0.001	2.36	1.90, 2.92	< 0.001
5	6.87	4.99, 9.46	< 0.001	6.84	4.96, 9.44	< 0.001	4.84	3.40, 6.90	< 0.001
≥ 6	9.5	5.46, 16.5	< 0.001	9.2	5.40, 15.7	< 0.001	7.25	4.23, 12.4	< 0.001
*P*-trend	< 0.001			< 0.001			< 0.001		

*CI* Confidence interval, *OR* Odds ratio

Model 1: adjusts for none

Model 2: adjusts for age, SEX race

Model 3: adjusts for age, SEX, education, race, BMI, PIR, cancer, smoking, alcohol, stroke, Coronary_heart_disease

### Subgroup and interaction analyses

**Fig. 2:**
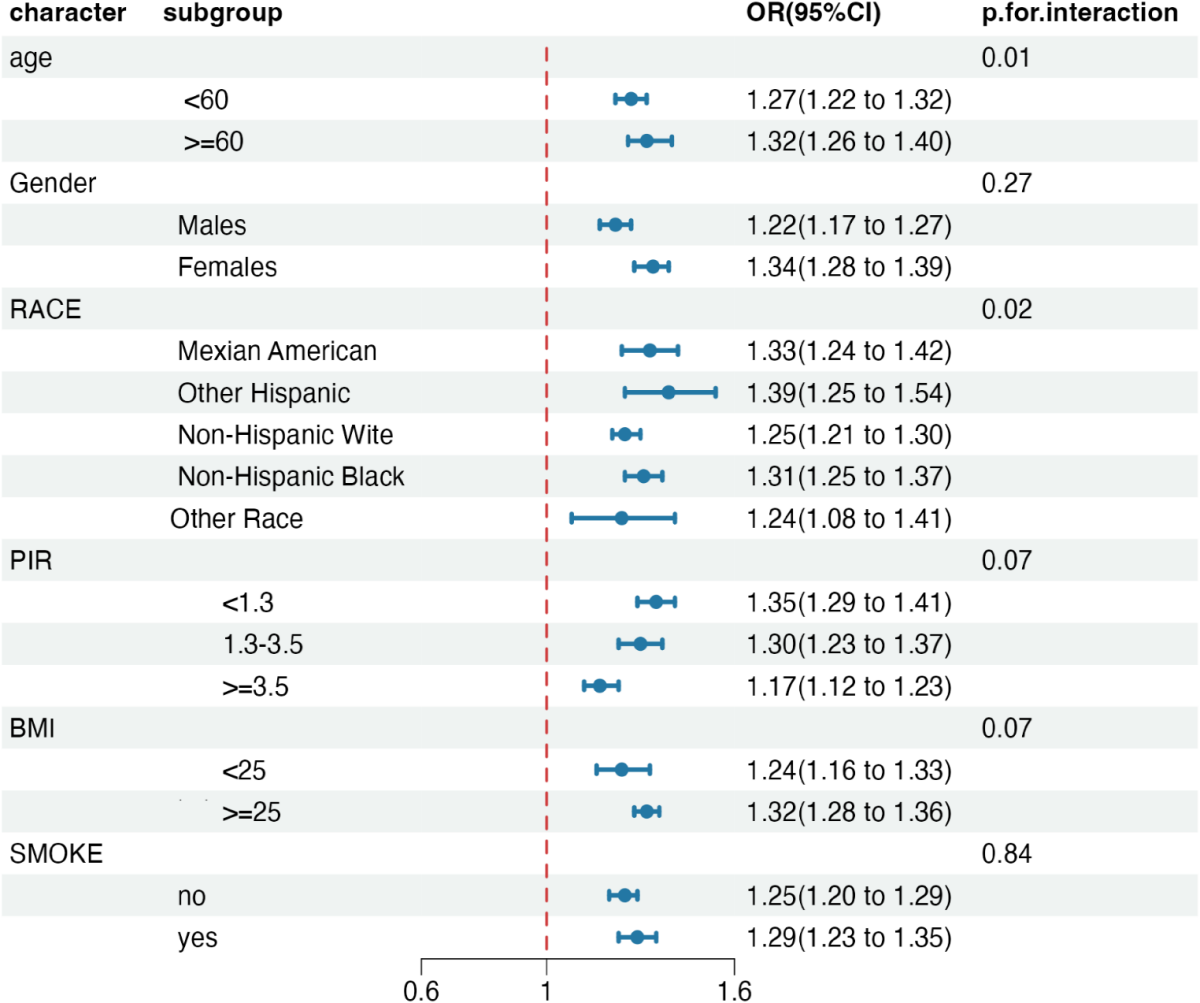
Multivariate regression models were used to analyze subgroup analyses and forest plots of the association between circadian syndrome and frailty, ratio of ratios (OR). The models were adjusted for the variables listed in the fully adjusted model

To further explore the relationship between Circadian Syndrome (CircS) prevalence and frailty, interaction analyses were conducted within fully adjusted models (refer to Fig. 2). Our findings revealed significant variation in the association based on age (*p* = 0.01) and race (*p* = 0.02). However, it is important to note that the direction of the association remained consistent across all subgroups. No other significant interactions were observed with additional factors.

### Sensitivity analysis

In conducting a sensitivity analysis, we matched data from groups with and without frailty for age, sex, BMI, education, and ethnicity. This analysis, detailed in Supplementary Table 2, reaffirmed the positive correlation between CircS and frailty. A key finding is that a higher prevalence of CircS correlates with an increased risk of frailty. Furthermore, a greater number of abnormal CircS components was associated with a heightened risk of frailty.

## Discussion

This cross-sectional study aimed to evaluate the association between Circadian Syndrome (CircS) and frailty among adults in the United States, utilizing data from six cycles of the National Health and Nutrition Examination Survey (NHANES). Our findings indicate a significant positive correlation between CircS prevalence and frailty. Notably, as the number of CircS components increased, so did the risk of frailty. This correlation was further accentuated by factors such as age and ethnicity, potentially reflecting variations in circadian gene expression or lifestyle habits across different population groups. To enhance the robustness of our findings, we conducted propensity score matching for groups with and without frailty, followed by a sensitivity analysis. These approaches consistently demonstrated the same trend. Consequently, we consider our findings to be highly reliable and indicative of a strong link between CircS and frailty in the U.S. adult population.

Previous research has predominantly focused on the separate links between frailty and metabolic syndrome (MetS) [9], and between frailty and circadian rhythm disruption [25]. However, a simultaneous analysis of these factors, crucial for understanding their interplay, has been lacking. Our study is a pioneering effort to investigate the direct association between Circadian Syndrome (CircS) and frailty. CircS encompasses a spectrum of psychological and physiological disorders, including MetS (central obesity, elevated serum triglycerides, reduced serum HDL-C, plasma glucose, and hypertension), depression, and alterations in circadian rhythms.

CircS may contribute to frailty through various mechanisms. Firstly, it can impact metabolic health; clinical studies suggest that the circadian rhythm changes commonly experienced by shift workers can hasten the onset of chronic diseases like cardiovascular disease, hypertension, and MetS [26, 27], which in turn elevate frailty risk [28]. Secondly, CircS is linked to psychological disorders. Elevated levels and severity of frailty have been observed in patients with psychological conditions such as depression and anxiety. Hoyos et al. noted marked circadian dysregulation in elderly patients with depression [29]. These psychological states can lead to reduced activity, nutritional imbalances, and other health complications, thereby fostering frailty.Lastly, CircS is closely associated with circadian rhythm disruption[25, 30], defined as abnormalities in the biological clock’s variables over a 24-hour cycle [31, 32]. Studies have shown that circadian rhythms are primarily regulated by the major biological clocks in the suprachiasmatic nucleus (SCN) of the hypothalamus [33]. Disturbances in circadian rhythms, such as diminished intensity, stability, or increased variability, are strongly linked with an escalated risk of developing frailty and its rapid progression [34]. About 50% of patients with sleep disorders, especially insomnia, suffer from anxiety, which can be triggered or exacerbated by sleep deprivation [35] .Anxiety and depression often correlate with disruptions in normal sleep-wake mechanisms [36], and circadian rhythm disruption may heighten anxiety and depressive symptoms, thus increasing frailty risk [37].

However, while our study provides initial evidence of a relationship between CircS and frailty, many questions remain unanswered, necessitating further research. For instance, the specific impact of CircS on metabolic, immune, and other physiological functions is not yet fully understood.

Correlation between Circadian Syndrome (CircS) prevalence and frailty, although the direction of this effect remained consistent across different subgroups. Circadian rhythm disruption is known to accelerate aging, and aging itself renders individuals more susceptible to circadian disturbances [38, 39]. Prior research indicates an increase in CircS prevalence with advancing age, and a clinical trial has highlighted significant elevations in circadian rhythm disorders in older populations [40]. Hence, while substantial efforts have been made, further trials and research are necessary to elucidate the underlying pathological and psychological mechanisms. The significance of maintaining physiological and metabolic health through normal circadian rhythm regulation is highlighted by this interactive relationship [41]. As individuals age, the regulatory capacity of the circadian clock decreases, making the circadian rhythm more susceptible to disturbances. This susceptibility not only affects sleep quality but may also worsen cognitive decline [42] and frailty. Furthermore, an interruption in the circadian rhythm increases the likelihood of patients developing behavioral and psychological symptoms of dementia (BPSD) [43]. It is worth noting that patients with BPSD often suffer from sleep disorders [44] ], including nocturnal awakenings and a reversal of day-night cycles. These disruptions not only increase the risk of delirium and cognitive impairment but may also directly indicate an imbalance in the circadian rhythm [45] Therefore, the patient population with BPSD offers a valuable perspective to investigate the correlation between circadian syndrome and geriatric frailty. Circadian regulation is a complex physiological process that affects multiple bodily systems [18, 46]. Any disturbance in the circadian rhythm can disrupt the harmonious functioning of these systems, hastening the aging process at the cellular and tissue levels, and thus impacting an individual’s overall health and lifespan [47]. Furthermore, as individuals age, there is a general decline in physiological functions, including reduced sensitivity to light and other zeitgebers. This decline makes the elderly particularly susceptible to circadian syndrome, which establishes a complex interplay between circadian syndrome, dementia, and aging.

### Study limitations

This study, utilizing the NHANES database from a national multi-ethnic survey, boasts strengths such as a large sample size, enhancing the generalizability and representativeness of its findings. While it is becoming increasingly clear that chronological age alone is not sufficient to comprehensively define the onset of physiological deterioration associated with aging, frailty indices stand out as valuable tools capable of detecting characteristic features of biological aging, assessing changes in intrinsic capabilities, and facilitating the development of effective preventive strategies. Additionally, the incorporation of CircS, aligning closely with physiological processes, offers a novel perspective in this research field. This is the first study to examine the association between CircS prevalence and frailty in older adults, paving new pathways for future research. However, several limitations warrant mention. The absence of repeated CircS measurements may limit our understanding of its variations and its association with frailty in older adults. Additionally, the reliance on self-reported sleep duration rather than objective measures might affect the accuracy and validity of our findings. The threshold for depressive symptoms in the PHQ-9 was set at 5, potentially identifying only mild depressive symptoms, and while the PHQ-9 is convenient and efficient, it may not adequately assess more severe depressive states. Frailty diagnosis, based on questionnaire responses, could be prone to recall bias, impacting diagnostic accuracy. Furthermore, the cross-sectional nature of this study precludes establishing a causal relationship between CircS incidence and frailty in older adults. Finally, a notable limitation is the use of a frailty index definition that assigns equal weight to each health problem regardless of its severity, such as cancer and abnormalities in body mass index (BMI). This one-size-fits-all scoring method, which assigns one point for each condition, may appear arbitrary or impractical because it does not account for the varying severity and impact of different health problems on an individual’s overall health. As a result, this scoring approach may inadequately capture an individual’s vulnerability by failing to distinguish between the severe consequences of critical conditions and the milder effects of less severe problems. The Multidimensional Prognostic Index (MPI), based on the Comprehensive Geriatric Assessment (CGA), is a well-recognized tool for frailty assessment [48].

However, its application is limited by the lack of multidimensional indicators in certain databases, such as the National Health and Nutrition Examination Survey (NHANES) database. This limitation hinders comprehensive assessment by the full MPI. Therefore, refinement of gold standards for frailty diagnosis is imperative to accurately capture the multifaceted nature of this condition and facilitate accurate identification and targeted intervention strategies for at-risk older adults.

## Conclusion

Our research identified a significant positive association between CircS and frailty in older adults. Moreover, the risk of frailty was notably higher with an increasing number of CircS components. These findings provide critical insights into the complex interplay between circadian syndrome and frailty in older age, potentially guiding the development of targeted preventive and intervention strategies.

### Electronic supplementary material

Below is the link to the electronic supplementary material.

Supplementary Material 1

Supplementary Material 2

## Data Availability

No datasets were generated or analysed during the current study.
