# Supplementary material for: The Association between Circadian Syndrome and Frailty in US adults: a cross-sectional study of NHANES Data from 2007 to 2018
Source: Aging Clin Exp Res. 2024 May 7;36(1):105. doi: 10.1007/s40520-024-02745-3 (PMC11076391; doi:10.1007/s40520-024-02745-3)
Supplement: Supplementary file 1 — Supplementary Material 1 [file 40520_2024_2745_MOESM1_ESM.docx]

| **Supplementary table2. Sensitivity analysis of propensity scores for the presence of frality both groups.** | | | | | | | | | |
| --- | --- | --- | --- | --- | --- | --- | --- | --- | --- |
|  | **model1** | | | **model2** | | | **model3** | | |
| Characteristic | OR1 | 95% CI1 | *p*-value | OR1 | 95% CI1 | *p*-value | OR1 | 95% CI1 | *p*-value |
| CircS | 2.68 | 2.37, 3.03 | <0.001 | 2.66 | 2.36, 2.99 | <0.001 | 2.18 | 1.91, 2.49 | <0.001 |
| Components of circadian syndrome | | | <0.001 |  |  | <0.001 |  |  | <0.001 |
| <4 | Ref | | | Ref | | | Ref | | |
| 4 | 3.02 | 2.45, 3.71 |  | 3.02 | 2.45, 3.73 |  | 2.44 | 1.94, 3.08 |  |
| 5 | 7.14 | 5.23, 9.73 |  | 7.16 | 5.25, 9.75 |  | 5.18 | 3.64, 7.39 |  |
| ≥6 | 12.3 | 6.15, 24.8 |  | 12.4 | 6.18, 24.9 |  | 10 | 4.84, 20.7 |  |
| *P* trend | <0.001 | | | <0.001 | | | <0.001 | | |
