# Supplementary material for: The Association between Circadian Syndrome and Frailty in US adults: a cross-sectional study of NHANES Data from 2007 to 2018
Source: Aging Clin Exp Res. 2024 May 7;36(1):105. doi: 10.1007/s40520-024-02745-3 (PMC11076391; doi:10.1007/s40520-024-02745-3)
Supplement: Supplementary file 2 — Supplementary Material 2 [file 40520_2024_2745_MOESM2_ESM.docx]

| **Supplementary table 1: Detailed scoring criteria for 49 vulnerability indices.** | |
| --- | --- |
| Physical Performance and Anthropometry | |
| 1. Body mass index | <18.5 or ≥30 = 1 |
|  | 18.5 to 25 = 0 |
|  | 25 to 30 = 0.5 |
| 2. Handgrip strength | Male: |
|  | For BMI 24 to 28, GS ≤ 30 = 1; |
|  | For BMI ≤ 24, GS ≤ 29 = 1; |
|  | For BMI >28, GS ≤ 32 = 1. |
|  | Female: |
|  | For BMI 23 to 26, GS ≤17.3 = 1; |
|  | For BMI>29, GS ≤ 21 = 1. |
|  | For BMI ≤23, GS ≤17 = 1; |
|  | For BMI 26 to 29, GS ≤ 18 = 1; |
| **Comorbidities** | |
| 3. Chronic bronchitis | No = 0 |
| 4. Thyroid problems | Suspect = 0.5 |
| 5. Arthritis | Yes = 1 |
| 6. Congestive heart failure |  |
| 7. Cancer |  |
| 8. Coronary heart disease |  |
| 9. Angina |  |
| 10. Weak/failing kidneys |  |
| 11. Heart attack |  |
| 12. Blood pressure |  |
| 13. Stroke |  |
| 14. Urinary Leakage |  |
| 15. Diabetes |  |
| 16. Experience confusion/memory problems | Yes = 1 |
|  | No = 0 |
| Hospital Utilization and Access to Care | |
| 17. Number of prescribed medications | None = 0, 1 to 4 = 0.5, ≥5 = 1 |
| 18. Overnight hospital patient in past year | Yes = 1 |
|  | No = 0 |
| 19. Health now compared with 1 year ago | Worse = 1 |
|  | About the same, Better = 0 |
| 20. Self-rated health | Fair or poor = 1 |
|  | Excellent, very good, or good = 0 |
| 21. Frequency of healthcare use during the past year | None = 0, 1 to 5 = 0.5, More than 5 = 1 |
| Dependence | |
| 22. Managing money | Difficulty = 1 |
| 23. Stooping, crouching, kneeling | No Difficulty = 0 |
| 24. Walking up 10 steps difficulty |  |
| 25. Lifting or carrying |  |
| 26. Walking for a quarter mile difficulty |  |
| 27. House chore |  |
| 28. Attending social events |  |
| 29. Preparing meals |  |
| 30. Standing for long periods difficulty |  |
| 31. Standing up from armless chair |  |
| 32. Dressing yourself |  |
| 33. Using fork, knife, drinking from cup |  |
| 34. Grasp/holding small objects |  |
| 35. Getting in and out of bed difficulty |  |
| 36. Push or pull large objects |  |
| Laboratory Values | |
| 37. Glycohemoglobin (%) | 0% to 5.7% = 0, >5.7% = 1 |
| 38. Segmented neutrophils percent (%) | 40 to 80 = 0, Other = 1 |
| 39. Lymphocyte percent (%) | 20 to 40 = 0, Other = 1 |
| 40. Red cell distribution width (%) | 11.6 to 14.6 = 0, Other = 1 |
| 41. Hemoglobin (g/dL) | Male: 13.5 to 18 = 0, Other = 1 |
|  | Female: 12 to 16 = 0, Other = 1 |
| 42. Red blood cell count (million cells/ml) | Female: 4.2 to 5.4 = 0, Other = 1 |
|  | Male: 4.7 to 6.1 = 0, Other = 1 |
| Depressive Symptoms | |
| 43. Trouble sleeping or sleeping too much | Several days = 0.33 |
| 44. Trouble concentrating on things |  |
| 45. Have little interest in doing things | Nearly every day = 1 |
| 46. Feeling tired or having little energy | Not at all = 0 |
| 47. Feeling down, depressed, or hopeless | More than half the days = 0.66 |
| 48. Poor appetite or overeating |  |
| 49. Feeling bad about yourself |  |
